# First detection of bat-borne Issyk-Kul virus in Europe

**DOI:** 10.1038/s41598-020-79468-8

**Published:** 2020-12-24

**Authors:** Annika Brinkmann, Claudia Kohl, Aleksandar Radonić, Piotr Wojtek Dabrowski, Kristin Mühldorfer, Andreas Nitsche, Gudrun Wibbelt, Andreas Kurth

**Affiliations:** 1 Centre for Biological Threats and Special Pathogens, Robert Koch Institute, Seestraße 10, 13353 Berlin, Germany; 2 Methodology and Research Infrastructure 2: Genome Sequencing, Robert Koch Institute, Berlin, Germany; 3 Methodology and Research Infrastructure 1: Bioinformatics, Robert Koch Institute, Berlin, Germany; 4 Leibniz Institute for Zoo and Wildlife Research, Berlin, Germany

**Keywords:** Infectious-disease diagnostics, Virology

## Abstract

Bats have been gaining attention as potential reservoir hosts of numerous viruses pathogenic to animals and man. Issyk-Kul virus, a member of the family *Nairoviridae*, was first isolated in the 1970s from vespertilionid bats in Central Asia. Issyk-Kul virus has been described as human-pathogenic virus, causing febrile outbreaks in humans with headaches, myalgia and nausea. Here we describe the detection of a novel strain of Issyk-Kul virus from *Eptesicus nilssonii* in Germany. This finding indicates for the first time the prevalence of these zoonotic viruses in Europe.

## Introduction

Bats have been identified as reservoir hosts of several highly pathogenic viruses such as Hendra virus, Nipah virus and SARS virus^1–5^. Numerous studies have been focusing on the identification of other highly pathogenic viruses possibly transmitted by bats, and a plethora of novel viruses was described^6^. Subsequently, bats are increasingly being recognized as potential reservoir host of other viruses (i.e. bunyaviruses) that may also have the ability to cause severe disease in humans. Within the order *Bunyavirales*, the family *Nairoviridae* contains the genus Orthonairovirus, named after the Nairobi sheep disease orthonairovirus (NSDV) species^7^. NSDV and other members of the genus, like Crimean Congo Hemorrhagic Fever virus, Dugbe virus and Ganjam virus, are highly pathogenic to animals and humans^8^. Orthonairoviruses are often transmitted by ticks, although the vertebrate reservoir host of the viruses remains unknown; the virus has not been found in wild ruminants or other animals in enzootic areas. A bat nairovirus, Ahun nairovirus, has been detected in lung tissues of one *Pipistrellus pipistrellus* and one *Myotis mystacinus*^9,10^. Phylogenetically, Ahun nairovirus appears as a new clade, distinct from other orthonairoviruses^10^. Another bat orthonairovirus, identified by metagenomics, originates from fecal samples of *Molossus molossus* bats from French Guiana^11^. In a comprehensive study the virome of European bats in nine pools was sequenced and, besides three novel nairoviruses, numerous viruses were identified^12^. However, in this manuscript we describe the further characterization of a novel Issyk-Kul virus strain PbGER (ISKV PbGER), from German bats by virome sequencing by annotation and phylogeny.

## Results (with subheadings)

Organ tissue homogenates from 12 bats (*Eptesicus nilssonii)* showing histopathological alterations were pooled and purified using the TUViD-VM protocol and subjected to virome sequencing^13^. The total read number after sequencing was 9,806,241 reads of which 7970 were specifically mapping to Issyk-Kul virus (ISKV), a member of the genus *Orthonairoviridae*.

The identified virus sequences shared the highest identity with the strain Issyk-Kul virus KR709221 and were confirmed by sequence assembly and comparison to Issyk-Kul virus reference strain KR709221. The virus was named Issyk-Kul virus strain PbGER (ISKV PbGER) after the origin of the bats in Prackenbach, Germany. Individual bats were tested back for ISKV PbGER with newly designed specific primers and confirmed by conventional PCR and Sanger sequencing in tissues of nine individual bats. To extend the ISKV PbGER L segment, primer walking was utilized. Initially the assembled sequence of the L segment was interrupted by 35 gaps. The longest continuous part of the sequence had a length of 1281 nt (supplemental section). In this study, we were able to reduce the number of gaps to 10 and recover 93.5% of the L segment with an identity of 95.1% nt to strain LEIV-315K (KR709221) by using primer walking and Sanger Sequencing. The longest continuous part of the L segment sequence is now 6154 nt long (MN851301).

**Figure 1:**
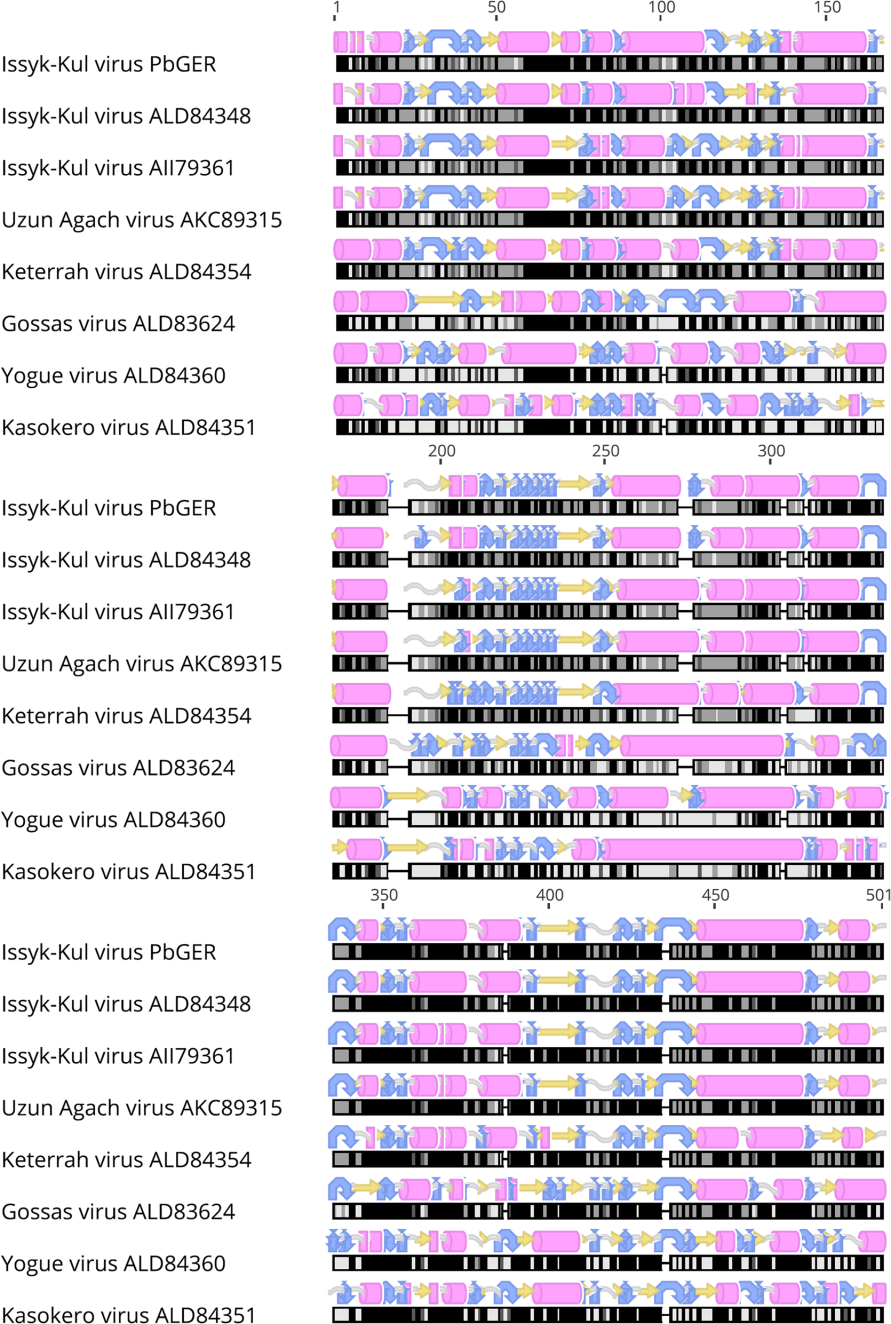
Protein alignment and secondary structure prediction of the nucleoprotein of Issyk-Kul virus strain PbGER in comparison with selected members of the orthonairoviruses.

**Figure 2:**
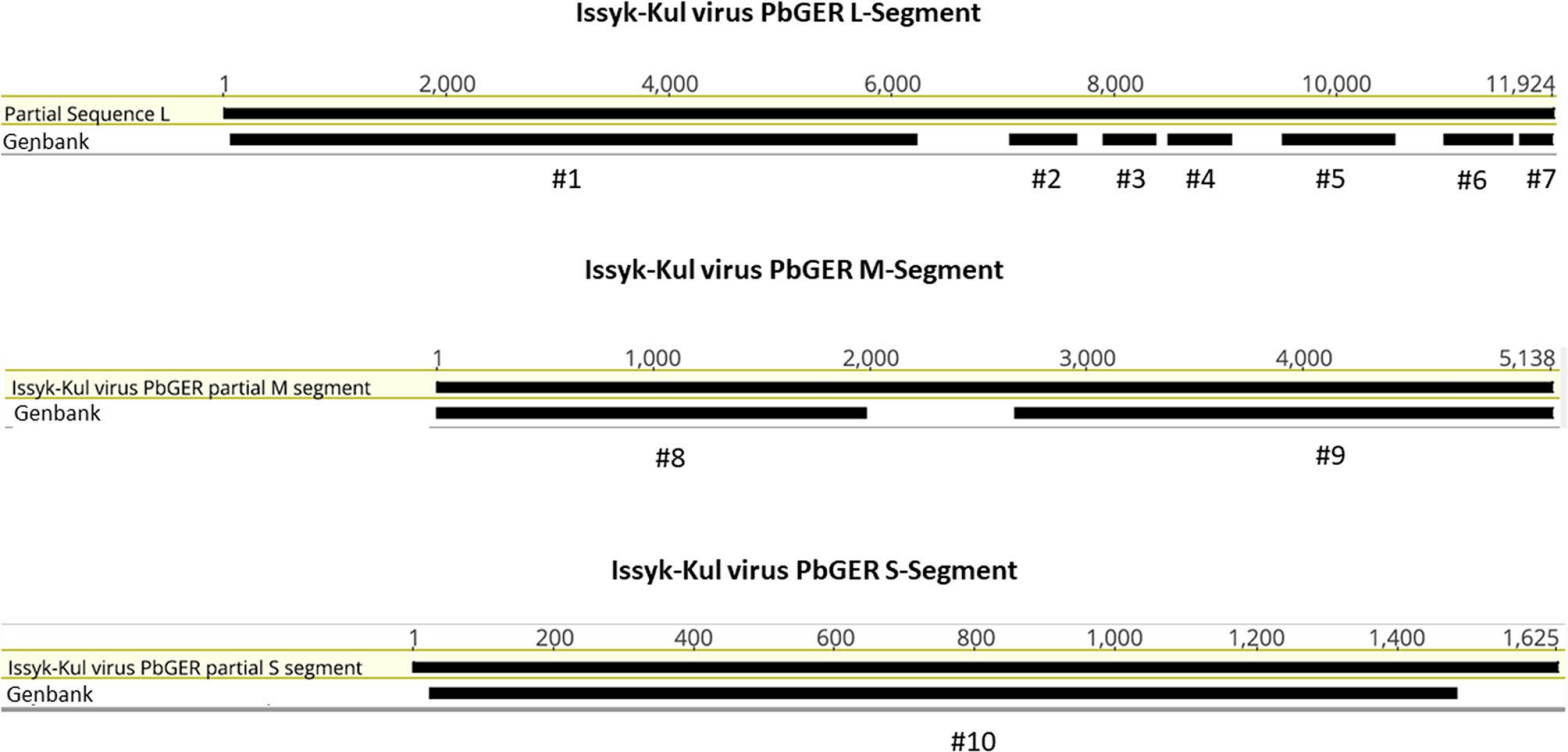
Allocation of available seuquences of Issyk-Kul virus PbGER on the respective segments.

Assembly of the M segment resulted in 89% of the segment length with 87% nt % identity to strain LEIV-315K (KR709221). For the S segment, 98% of the whole length and an identity of 89.2 nt % with LEIV-315K (KR709221) was reached (compare with Fig. 1). Due to limited sample quantity further extension of the sequence was not possible. Table 1 and Fig. 2 are giving an overview on available sequences, their accession numbers and location on the three segments. Further the supplemental section contains the obtained sequences after assembly and the final sequence of the L segment. A further real-time PCR was developed to estimate the viral loads in the individual organs. The lowest CT values were obtained in Bat 194/07 liver, spleen and lungs. Results of the real-time PCR screening are summarized in Table 2. All available Accession numbers are listed in Table 1.

**Table 1:** Issyk-Kul virus PbGER sequences uploaded to NCBI Genbank with length and identity to Issyk-Kul-virus strain LEIV-315K.

Partial sequence	Name	Length (nt)	nt (aa) % ID with LEIV-315K KR709221	Accession no
#1	Issyk-Kul virus PbGER L segment partial 1	6142	96.7 (99.3)	MN851301
#2	Issyk-Kul virus PbGER L segment partial 2	579	96.4 (98.9)	MW275288
#3	Issyk-Kul virus PbGER L segment partial 3	446	96.8 (98.0)	MW275289
#4	Issyk-Kul virus PbGER L segment partial 4	553	94.2 (98.9)	MW275290
#5	Issyk-Kul virus PbGER L segment partial 5	981	95.6 (99.7)	MW275291
#6	Issyk-Kul virus PbGER L segment partial 6	588	94.7 (98.5)	MW275292
#7	Issyk-Kul virus PbGER L segment partial 7	271	97.6 (100)	MW275293
#8	Issyk-Kul virus PbGER M segment partial 8	1968	82.0 (79.4)	MW275294
#9	Issyk-Kul virus PbGER M segment partial 9	2471	86.1 (97)	MW275295
#10	Issyk-Kul virus PbGER nucleoprotein complete	1458	83.1 (96.5)	MW275296

*nt* nucleotides, *aa* amino acids.

**Table 2:** Results of screening for PbOVby conventional and real-time PCR.

Sample identification		Organs used for Virome								Results PbOV	
Bat ID	*Species*	Lu	Li	He	Ki	Sp	Br	SG	In	cPCR + Sanger	qPCR results (Ct)^a^
E 108/09	*Eptesicus nilssonii*	1		1		1	1		1	Sp, Lu	Lu 38. Sp 38, In 38
E 138/09	*Eptesicus nilssonii*	1	1			1			1	Li, Sp, In	Li 38, Sp 37, In 38
E 139/09	*Eptesicus nilssonii*	1	1			1				Sp, Lu	–
E 141/09	*Eptesicus nilssonii*	1	1			1				N	n/d
E 143/09	*Eptesicus nilssonii*	1	1			1				Br, In, He	Sp 37, Br 40
E 145/09	*Eptesicus nilssonii*	1								N	n/d
E 147/09	*Eptesicus nilssonii*	1				1			1	Lu	–
E 148/09	*Eptesicus nilssonii*	1				1				Sp	–
E 193/07	*Eptesicus nilssonii*	1				1				N	n/d
E 194/07	*Eptesicus nilssonii*	1							1	Lu, Li, Sp	Br 29, Lu 25, Li 19, Sp 37
E 196/07	*Eptesicus nilssonii*	1								Li, Sp	Lu 36, Sp 37
E 202/07	*Eptesicus nilssonii*					1				Li, Lu	Sp 38

*Lu* lungs, *Li* liver, *He* heart, *Ki* kidney, *Sp* spleen, *Br* brain, *SG* salivary glands, *In* intestine, *cPCR* conventional PCR, *qPCR* quantitative PCR.

^a^qPCR values represent the copies/µl: ranging from Ct 40 ~ 1 copy to Ct 19 ~ 10 million copies.

**Figure 3:**
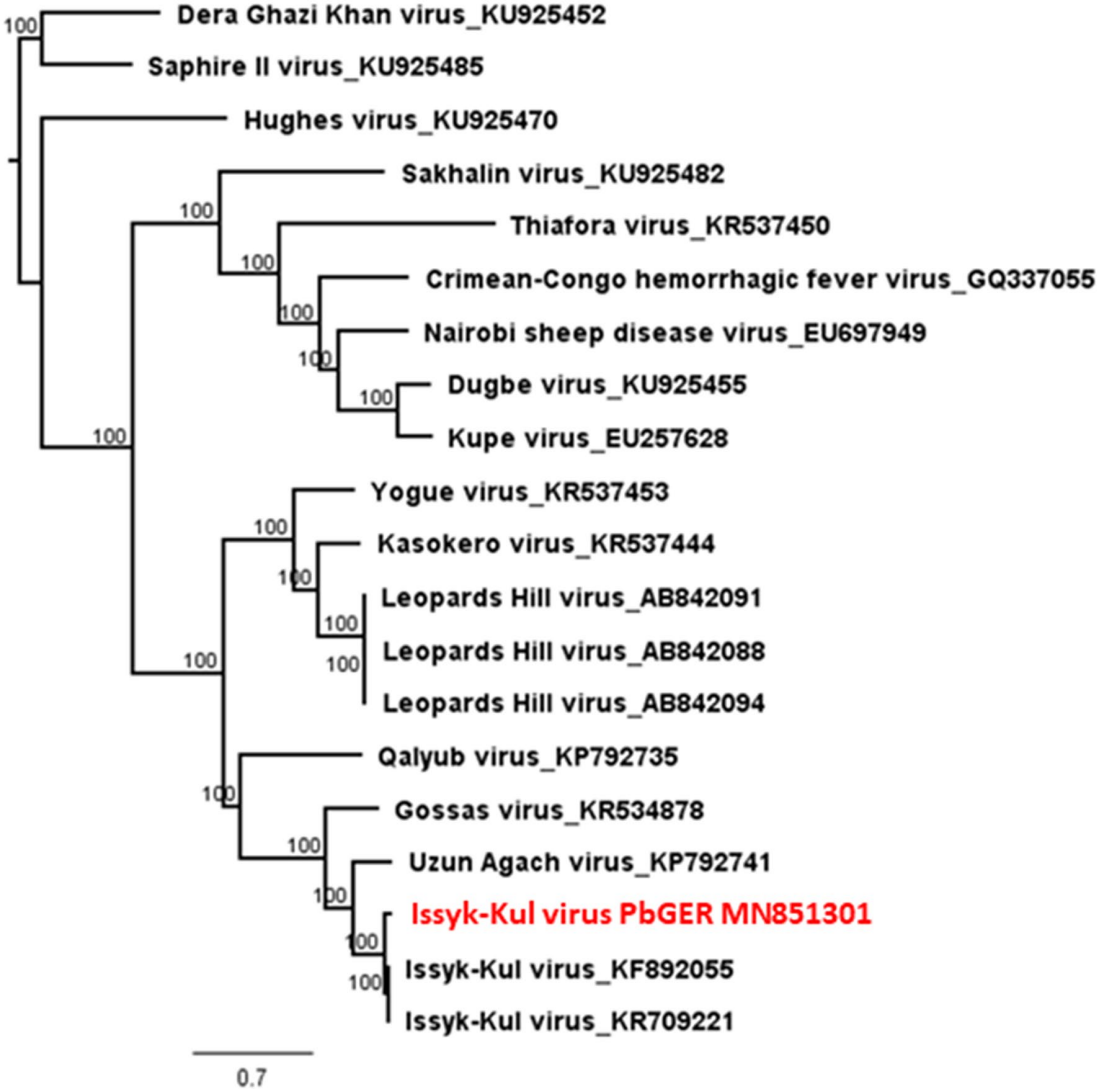
Phylogenetic reconstruction of ISKV PbGER (L-segment 6650 nt) with other members of the Keterah group and of the family *Nairoviridae*.

Phylogenetic reconstruction of ISKV PbGER with other members of Orthonairoviruses (6650 nt, L-segment) is displayed in Fig. 3. The annotated proteins were similar in structure and length to proteins from the reference strain Issyk-Kul virus. 2D structures of ISKV PbGER nucleoprotein (segment S) in comparison to other strains of the Keterah group display further evidence for grouping ISKV PbGER into this group (available on request).

## Discussion

The majority of nairoviruses is transmitted by ticks several of which are capable of causing severe disease in humans and animals^14^. In this study a novel nairoviral (ISKV PbGER) sequence was detected and further characterized. ISKV PbGER shows the highest identity with Issyk-Kul virus strain LEIV-315K (Id 95% nt; 99% aa). Nine out of twelve *Eptesicus nilssonii* bats sequenced, proved to be infected with ISKV PbGER (Table 2). The phylogenetic reconstruction clearly allocates this novel ISKV strain to the already described clade of Issyk-Kul viruses within the Keterah genogroup (Fig. 3)^9^. Issyk-Kul virus was first isolated in 1970 from a *Nyctalus noctula* bat in Kyrgyzstan and later on in Tajikistan and Kazakhstan^15,16^. Issyk-Kul virus has been described to cause sporadic febrile outbreaks in humans with headaches, myalgia and nausea^17^. It is assumed that Issyk-Kul can be transmitted by tick bites and exposure to bat feces and urine^17^. The ISKV PbGER virus described here was found predominantly in liver, spleen and lung tissues of the respective bats, indicating systemic infection of bats instead of merely passaging of intestinal tick content. The protein alignment (Fig. 1) of the nucleoprotein in comparison to other members of the orthonairoviruses further supports the results of the phylogenetic reconstruction. In Fig. 1 the Uzun-Agach-virus, a virus also isolated from bats (*Myotis blythii*) in Kazakhstan, is very close related to ISKV PbGER^18^. This was also seen for the L segment in Fig. 2. The authors claim that Uzun-Agach virus may have resulted from reassortment with Issyk-Kul virus. This underlines the abundance of these viruses in bat population throughout Central Asia and Europe.

Issyk-Kul virus is a known zoonotic virus described already more than 40 years ago in Central Asia. Our findings show for the first time the abundance of this virus in Europe and within the species, *Eptesicus nilsonii*. *Eptesicus nilsonii* is a common bat distributed throughout Asia and Europe (including the polar regions). In Scandinavia they are even the most frequent bat species^19^. They are dependent on humid habitats in close proximity to fresh water. In winter, they hibernate on heated attics and wall claddings of human dwellings. Issyk-Kul virus is described to be transmissible by bat urin and feces. Taking all this information’s into account, there is a potential for bat to human contact.

Furthermore, these findings indicate that the described ISKV PbGE(R from *Eptesicus nilssonii* might also have the potential of zoonotic transmission to humans and should be subject of further investigation. Our findings support the theory that bat species may share a long evolutionary history with orthonairoviruses and may have continuing cycles of transmission because of preying on varying tick species.

## Methods

### Study

Since all German bats are covered by species protection through the European Commission (http://ec.europa.eu/environment/nature/legislation/habitatsdirective) and through the Agreement on the Conservation of Populations of European Bats (www.eurobats.org), investigative research requires special permission by local government bodies^20^. We herewith confirm, that all permits to investigate carcasses of deceased bats and all experimental protocols on the dead bats were approved by the respective local governmental authorities (district government of Upper Bavaria, Munich [No. 55.1-8642.1-4-2006]; district government of Bavarian Swabia, Augsburg [No. 51-8645.11/489]; Lower Saxony water management, coastal defence and nature conservation, Hannover [No. 897.21-20]; senate department for urban development and the environment, Berlin [No. I E 222-10.04.2004]). We herewith further confirm that all experiments were performed in accordance with relevant guidelines and regulations. As part of a study on diseases in native bats^20^, organs of 12 *Eptesicus nilssonii* bats showing histo-pathological alterations were examined. The pool consists of the following individuals and organs (Table 2).

Several bats were found to have alterations that may be related to viral infections (e.g., interstitial pneumonia, pulmonary BALT hyperplasia, infiltrates of mononuclear cells in different organs, diffuse enlarged villi and mononuclear intestinal cell infiltrates, catarrhal or hemorrhagic enteritis). Animals were found dead, injured or moribund near roosting sites or human habitations^20^ in urban and suburban areas in Bavaria, Germany. All bat carcasses were kindly provided by bat researchers and bat rehabilitation centers from the different geographic regions. Permits to investigate carcasses of deceased bats were granted by the respective local governmental authorities (district government of Upper Bavaria, Munich, Germany). If bats died in care or had to be euthanized for medical reasons, the carcasses were immediately stored at − 20 °C and transferred to the Leibniz Institute for Zoo and Wildlife Research, Berlin, Germany, for pathological and bacteriological examination^20–23^. Aliquots of the individual organs were divided between one tube with RNAlater® (Qiagen, Hilden, Germany) for RNA extraction and one tube native frozen at − 80 °C and sent to the Robert Koch Institute for virological examination^12^. Samples were pooled and viruses purified using the TUViD-VM protocol and subsequently prepared for sequencing. Libraries were built as sequencing was performed as described before^12^.

### Data analysis and confirmation

Raw data was processed as described before^12^. All work was performed at Biosafety level-2 conditions with appropriate precautions. After obtaining the result that the novel strain is Issyk-Kul virus (rated BSL-3 in Germany), further work on the original material was restricted to BSL-3. Homogenized organs [n = 31] with potentially virus-related histopathological changes (data available on request) were pooled (Table 2), followed by purification and enrichment of viruses directly from virus-infected tissue and subsequent deep sequencing of the virome^11,12^. Sequences of highest interest were extended by spanning PCRs and primer walking followed by Sanger sequencing. For sequences of interest specific primers were designed to confirm the sequences in cDNA by conventional PCR of the individual extracted pools and organ samples eventually. Cycling conditions and primers are available on request. Any bands of targeted length were purified (MinElute PCR Purification Kit, Qiagen), Sanger sequenced and compared to the original sequence obtained from NGS. For ISKV PbGER a real-time PCR was designed to test the individual organs of all *Eptesicus nilssonii* bats involved in the initial study. Primers, probes, mastermix and cycling conditions were as follows: ISKV PbGER-F (5′-GTGGAAGAATAACGCACCCCATAT-3′), ISKV PbGER-R (5′-ATGCACCAGGTCGTTTGTGGT-3′), Probe: ISKV PbGER-P FAM-TCCACTGAAGGCTGGTAGGA. The mastermix consisted of 10 × buffer (Platinum Taq Kit), 1 µl of dNTPs (2.5 mM, Invitrogen), 0.75 µl of primer F (10 µM), 0.75 µl of primer R (10 µM), 0.25 µl of probe (10 µM), 3 µl of MgCl_2_ (25 mM, Platinum Taq Kit), 14.55 µl of H_2_O (Platinum Taq Kit) and 5 µl of cDNA. Cycling conditions were 600 s at 95 °C followed by 45 cycles of 15 s at 95 °C and 30 s at 60 °C.

### Phylogenetic reconstruction

Final sequences obtained from data analysis and Sanger sequencing were used to reconstruct phylogenetic trees. Sequences were aligned to type species of the respective viral family (ICTV database) and other related viruses of interest via ClustalW. Alignment quality was checked with the online tool T-Coffee^24^. Model of evolution was predicted via JModelTest and the model with the best AIC score was picked^25^. The actual reconstruction was performed via the Bayesian MCMC approach using MrBayes (burn-in 30%; frequency 100; chain length 1 million to 10 million depending on when a standard deviation of below 0.025 was reached)^26^. Reconstructed tree was visualized using FigTree and posterior probabilities were depicted for each node (http://tree.bio.ed.ac.uk/software/figtree/).

## Supplementary Information

Supplementary Infomations.
