# Supplementary material for: First detection of bat-borne Issyk-Kul virus in Europe
Source: Sci Rep. 2020 Dec 24;10:22384. doi: 10.1038/s41598-020-79468-8 (PMC7759570; doi:10.1038/s41598-020-79468-8)
Supplement: Supplementary file 1 — Supplementary Infomations. [file 41598_2020_79468_MOESM1_ESM.docx]

**Supplement to**

**First Detection of bat-borne Issyk-Kul virus in Europe.**

Annika Brinkmann^1*^, Claudia Kohl^1#*^, Aleksandar Radonić^2^, Piotr Wojtek Dabrowski^3^, Kristin Mühldorfer^4^, Andreas Nitsche^1^, Gudrun Wibbelt^4^, Andreas Kurth^1^

^1^Robert Koch Institute, Centre for Biological Threats and Special Pathogens, Berlin, Germany

^2^Robert Koch Institute, Methodology and Research Infrastructure 2: Genome Sequencing, Berlin, Germany

^3^Robert Koch Institute, Methodology and Research Infrastructure 1: Bioinformatics, Berlin, Germany

^4^Leibniz Institute for Zoo and Wildlife Research, Berlin, Germany

*contributed equally

^#^corresponding author:

Claudia Kohl

Centre for Biological Threats and Special Pathogens
Robert Koch Institute
Seestraße 10

13353 Berlin, Germany

[kohlc@rki.de](mailto:kohlc@rki.de)

Keywords: virome, metagenome, bats, bunyavirus, nairovirus, Illumina, HiSeq, virus discovery, TUViD-VM, chiroptera, Issyk-kul virus

LOCUS Partial_Sequence_L 11924 bp DNA linear UNA 12-NOV-2020

DEFINITION A new nucleotide sequence entered manually.

ACCESSION urn.local...cs-cn36sug

VERSION urn.local...cs-cn36sug

KEYWORDS .

SOURCE

ORGANISM .

FEATURES Location/Qualifiers

ORIGIN

1 ??ataagwgw ywkggagaat atagcttggg agcaggtcac tcaggagttc agcacggctg

61 tttgtaactt ccccatagag gaagttttta acatcgaccc catgctacct gatggcagat

121 gtttcttccg agctatggcc aagttcatgt tcaactcttc tgatgaatgg ctcattgtaa

181 agcgcgcttg tattgagtac tctcggcaac actggaacag gtttcttgtc tacactagac

241 tttatcctag ctctgctgat tatgagaggg acatcatgag ggatgactac tggggaggtt

301 ctttggaagc agaagtcctg tctgatttgt acaatctgac aattcacttc tgggtcacag

361 atgaccatca gtggatacac catgttcaaa ggtggaagaa taacgcaccc catatgagca

421 tcaatctgct attcaatcag aaccactttg acctgctaaa cctgaaagaa tttgaagagg

481 atgaggaaaa cctccctata ctacctttgg aggagaaggt ggatattgct ataagatctg

541 ttgtaacaga ttcagaagat ggttcagagc catctttctt aagagacatc tctgttgacg

601 actcaacctc taccttcaaa agaaatttag acttggagtt aatggaaatg gcaaatatga

661 aagagctact tttgaccaaa aaggggaata aaagaggttc caaacaaact gttgacatga

721 aagagaagga ttttaggaag gcagttgaaa aggggaagca aattccactg aaggctggta

781 ggattttaaa tcatctcttc agcattcaga tagaaggtgt aaatgaagga gaactcctga

841 ttctctaccc taaagacttt aaccacaaac gacctggtgc attctcaatt actgaccttg

901 gacacaagtt attggatggt agcaaagaat ttgctaaatc cattagcaaa ctaacacttg

961 tgatctctgg agagctgatg acgtacttaa atagcagtta cttaattagg atgtgcttcc

1021 caggcactgg tctcagtcaa actcctgatt tactacaccc tgctattaag attgacgcaa

1081 ctatcttggc atgcgcagtt cttattagct cctttcttta caagagttcg aatgacatta

1141 agaggaaatt ccttactctt gcttgtgaaa gaatgggtat caacacatca aagcttttca

1201 aagaaatgct taacttctgc aatactgaaa tgtatgagag tctttacaag gtagttcaac

1261 atttgtcagg agttttcttg aggaaacaag tggaagaggt ttcggagttt ttaaagcgta

1321 tgggcccgca gagcaaactg gcactacagt gcatcaacct tgagtcctgt gatcttaagc

1381 aatataagaa gttacttcag gaactaagtg aaagagatat gaatgatgta gactttaatt

1441 ctgaagaaat aaaggacctg catgaatgca tcgagcttat atcaaagttg tctaatgcgt

1501 caaaaccaaa tgctgacatc aaatctgaag tcagacagta ttgtacacat gttcccaaca

1561 agaaaaagtt ggaaagaggc aatattaagg atctcattgg gttgtgcatt attgaattct

1621 ttatgaggaa aatgattttt aaatttgtga gcctacaagg taaggcttat tcaggtgcct

1681 cccttggaaa tttgttagca tacgcacaca atctttactt gtcgaaagat agtcttaatt

1741 taactattga agatttaagc caactcgatg ttgagataag aagactcaac actctaatgt

1801 cagtagatgt aaagaaacca gttgcactta tttgcatgga gttagagaga aagttcacaa

1861 ggctgtttga acagctacct gaagattgta aacatgaatg tgaaacctta tttacagaca

1921 ttagaaatgc tgaaagtcat tcaagtgctt ggagatctgc tctgcggcta aagggaacag

1981 cctatgaggg tcttttctca aaacactata attggtcgta caccccagaa gaccttaaac

2041 cctcactagg tatgtcaatt cagacattgt tcccagaaaa gtttgagatg tttctggaaa

2101 gaacacacct acacccagaa ttcagggatt tggtgcctga cttcttttta tataaaccaa

2161 ggataattaa ggaggataca ttcgaagcat caataacgag gaaagatgat gatcaagtaa

2221 agcatgttga ccatattgag gctgtagaag acgcatcaac agtcaaactg acttcaaaga

2281 aaagatttcc attacctgaa gtaaacatac aagaagtctt atcaacacat aacttgtata

2341 aaggttttga gacaagagga agagagaaaa acaggcctat tttaacgaaa tcaacagaac

2401 aggatgcaag taatgatgaa gaggtcaaag ttaatgaact atgtctaatg gctgttgagg

2461 tcgggtatca gactgatgtt gaaggcaaag taatatcaga tatgaaaaaa tgggacagtg

2521 tgataagact aatgaaacac ataggtgtta actttagtgt cgtagcttgc gctgacagca

2581 cgaatacacc taaatctgac tggtggattc cggaagacat ggttcaactg ttattaaatt

2641 ccatcagtca cttatttaaa gaactgcaag agaattctcc tgttgatgtt acagatatag

2701 ctgttggtaa cataagtaca caaaaagtta gaagtgtctt gaggtcaggt gccatagtta

2761 aaactccagt tacctttaag gaaattaccg aaacatggca tgtcctaaag gaacatataa

2821 tcaacagacc tacaggagct gtgttggaca agttcacaga aaatgcaatg gaagcttcac

2881 ttgttgaggg cgcaatactc acaagagagt ctgcagagga tatattggag tatgtattag

2941 agaacatgga taaaataata ggtgagatgc aaaagaccaa atttaaacac gaaataagca

3001 agaaccagag gtctgcattt aaattattga ttggctggat gaatgaggac atactgagtt

3061 acagatgcaa tgactgtctc aagtcaatcc agagcaatat tagcaagata gaaactgaag

3121 ctgaagttgc atcttactta gcacgggagc tgatgccagt ggaaaaggat tgctgtaaaa

3181 gtatgcctca tacttatgaa gttagctctg tgcagagaag aatccctagc ctaacaaaca

3241 tcaaacacaa gccacttgtt gttaacgaga acaaaaccga aaaggatgga ggttcaacag

3301 tattggacag cttagttacc ctgacgttac ctggtaaaac agagaaagaa aggaagataa

3361 aaagagctgt tgagcagttg ataagagcta taatgagaca ttctagcctt ccagcaatta

3421 aactaccaag tgggcatcta ctactggacc acaacttaaa cagaccaata tatgatgaag

3481 agaatgaagg taaaagtttt aaaaagggtg gtcttaagtc cagagaggaa gatatggaac

3541 atttcaagaa acttctttca gaaagtaagt tatcttcgta ttctgaacac accaaacatg

3601 tgatccttgc tgctataaac aacttaagta actgcaaagg ggctaaatgc agtgttagtc

3661 agatgtgggt taaaaacata ctttacgact taaaatctga cacatctgat gaagttatta

3721 tgcaaaaggt tcaagaaagc tatgaaaaga agcagaactt ttctaatgaa agaaaattta

3781 aatatagacc tataggatgg tcccagataa aagattacct gctaagtaag aaagaactct

3841 atggtagtat gtccgttcca gtcttttcat tggattgtat actttttaag gaagtctgtc

3901 atgaagtatt aaggagactt gctaacactc cttatcagcc atgcatagat tacatagttg

3961 agctacttaa gttgcttctc aagtttcaat ggtaccagga gttagtctac tacggcaaag

4021 tgtgtgaaac tttcttacaa agttgcagtg aatttcatag atcaggaatt aaggttctaa

4081 ggataaggca caccgatacc aacttagtta tagcgctacc tgctaacaaa aaacagaata

4141 tgagatgttg tatctattct aaagatttct ccttaatcaa aggtccattt atgttgaaca

4201 ggagacaggc tgtcctaggt gcagcatatt gttatataat tccaatatgt tttctacagt

4261 gtctgcaaca ctacaggtgc gtttcagaac tagagtcgtt agatcaaaat attatgaaag

4321 acattctaag aagatctgaa cagcttcaca acctactatt agaaactcta gagatgactt

4381 acaatggtgt ctttgaaaca gcaaaaacga cacttttaaa tttttgcaaa aagtcaggca

4441 atttcatgac aagagggaca agggatcagt tcatctgctg ctttagtggt ctgagtgtaa

4501 catttagcac cttactaggt gatagtcttc ttaacaactc acagccattc aacaagcaga

4561 tccagatgat gaggtttgga ctgctcagtg ctatcagtag actgaactgt cctaaagaat

4621 taggaaagaa gttttcaagc agttgcagga aggtagaatt ccatgtttca aagctatata

4681 tgcagcttgt tgttttctct gccaactaca atgttgagac taattgtgaa aactggctca

4741 agactgatct ttgcagtcaa gctgaattgc cttgcttttc aatttttgga atgtttataa

4801 acagtgacag acaactaata tttgacattt accttgtaca tatctataat aaggagatgg

4861 atgactttga tgaagggtgt attaaggtgc tagaagaaac agctgagaga catgcagcat

4921 gggagataag tctcaaacga aatttagagc ttagtcagaa aagacatcag aaggataaat

4981 ctaaggataa agttttattc caagatgaca gtgcaaaaga agccaagctg gctcaacgca

5041 gagtcaggct cctgttaggc ttgccaaaca tcaaaaaaat gtctgaatct tatgttgact

5101 caagagatgc agaggaagaa gaagacttga gttcaagttc taaaactagc tcaacgatga

5161 gttacacttc ccgaaacagt aaatccagca ttaaaagtgc taggtcattt gcaaaaagga

5221 ggaacccgcc cacttctatg ttcggcataa gggcctcaaa acagaaacca atgagtatag

5281 agagcgggtt tgtcataact agggatgaca aaagagatta tcaacaagca ataacagaca

5341 aaggacttta tcatgagtac agagcaaata aagaatcagt ctttaaagac atcatcataa

5401 taataagaga gaacccaaac cacccctttg gcagctttga gctaatacaa gcatgtacag

5461 aaatagctag agcaaagttt cctcctgaag ctatagacaa agccaaaagg gatcctaaaa

5521 attggatcag tgtatcagaa gtaactgaaa caacaagcat cattgcggaa ccaagagatt

5581 ttatattcat caaggatgca tacagaatta ttataggcaa tgaaaataaa aaaatggtaa

5641 agctattaag aggaaagttt caaagactag gcctgtcttg taaatctgaa ggtcatgaca

5701 gggttcattg ccaagaatta ctctccacaa taccatcatt gacagacaag caaaaagatg

5761 atattatcaa aggtataatt aacccttcca aacttacatt ttacaattgg caagaactca

5821 tcaaaaaagg tgtgaatgaa gtgttgttra ctaatgatgg caattacata ttttgttggt

5881 taaagtcctt gagtcagatg gttaaaagtg gtttaagagg agaaataaag aatttaaagt

5941 acggacctat gttgcaaaaa ggcaagttgt gtcctaaaag taagatcttg agtagcgagg

6001 aacacagtgc cattaagaga tttattgagt ttttgaagtc atgtacaaag kgagaggtaa

6061 cagatgatat aaacaagtct gatataagta taacagatct aatcttggct tgggtgaaat

6121 tcacaaagtc ctccaaatta agcaagacga tcatctnnnn nnnnnnnnnn nnnctaagaa

6181 acctgtcaga aaagttgaaa aaacttgatc aagattataa tacactaata actttgaaga

6241 aagagcttcc tggtataagc ttttctaaag aagaaatact tcttagacag ggtgaaaaaa

6301 tgcttttgtt ggctcatgac aaagacataa tgcacctaac taacttgctc ttcttaatct

6361 gcttaagctg tccatggtgc attcagtaca agacttttga agctataatg atgaggaata

6421 tggctgaagc tgaaggattt aacttgccaa aaagtggaac taccattaat gagttacacc

6481 cagrytctgt tataaacatc cctggtaaaa gag??????? ?????????? ??????????

6541 ?????????? ?????????? ?????????? ?????????? ?????????? ??????????

6601 ?????????? ?????????? ?????????? ?????????? ?????????? ??????????

6661 ???gttaagc atataatggc ggtaactgga ttnnnnnnnn ctcgatctga ctttaaatgg

6721 actatcaacc tacttgccaa tagcaacttc gaggtgacta agaaaataac aggtagaagt

6781 gtgggtgaga agttgcctag gagtgttaga agtaaagtta tctatgaagt tgttaaacta

6841 gttgacaata ctgagatggc tatattgcaa caactgtctt tcacttatat actagatact

6901 aaccacagat tctttgccgt tcttgcaccc aaaagggcac aaagtggggt ggacataggg

6961 gatttttctt gtgcaggaaa acagggtagt aagatgatac acgccacaac tgagatgttt

7021 aagcagaacc ttgcttagca cttcgaaaga tgatgggcta acaaacaggc ggcttgaagg

7081 aatcaatact caatgcgggt ttggaggcca ttaacaccat gaagcttaac catggtaaag

7141 aaacttatcc tcagtcaggg cagtttcagt tctacaaagt gtggtgcata tctggtgata

7201 atactaaatg gggtccaata cattgttgtt cactgttcag tggaatgatg caacagcttc

7261 taaaggacat aaatgactgg tcctgttact ataaactaac cttcttaaaa aacctctgta

7321 ggcaagttga gattccttca tcttcaatta ggaaaatact aaattcattc aagtataaaa

7381 acagtgacgt taaagttgat gaacttccag aagatgagct gagggatatg ctgttccaaa

7441 gaatagacac ttggaatgat aatgagataa taaaattctt ggtggctaac tacatctcaa

7501 aaggtaaaat ggctataaac tcctacaatc atatgggtca gggtatacat catgcaacct

7561 catcagtgct aacatctata atggctcata ttatagagac actgataaag agatatttta

7621 agaaacacat gcctgactta g????????? ?????????? ?????????? ??????????

7681 ????gtgcat agttgctttt ggggtgttaa acagagcact ctacaaccat tatgaagaga

7741 gcttctggga gcacatgtgc aggctgaaga atttaatttc aggattttca agagcttgcc

7801 agatgaaaga ttcagctaaa acacttgttt ctgactgctt cttcaagttt tacagtgagt

7861 ttatgatgtc acaaagaata actccagctg ttataaaatt ccatattaac aggactcatt

7921 aatagctcag taacatcacc tttgagcctt atacaagctt gtcacgtctc tagtcaacag

7981 gctatgtaca atagcgtgcc attggtgaca aatcttgctt ttacactatt caggcagcag

8041 atgttttaca accacactga aaattttgtt agaacctatg gccatttaac attaggttct

8101 gtgtcaagct ttggtaggct gtatgtgccc aagtttagta atctcattgg ttcatcagtt

8161 gcacttgaag atgctgaaga aattacaaaa gcagctaaca atttaataaa agcaagtgtg

8221 cacttccctg aagcaattgg agacagctct ccatctagca agacatctga tgacagtgac

8281 tcaatgtata acgccaccac agattcaggt acagaagaca catcatctat tggtagtggn

8341 ?????????? ?????????? ?????????? ?????????? ?????????? ??????????

8401 ?????????? ?????????? ?????????? ?????????? ?????????? ??????????

8461 ?????????? ?????atgtc gtatttggac aatggtgact ttagcgaaga ttgttactac

8521 tcaaagatyc tggagtcata tagcgtcaag aacaatgact atcttggaga tgatgagagg

8581 tcaccagaac tagtgttgat gctgctgagg tctttgttga ttgtcttgat ttctggttac

8641 tacaggactt ttgccagtga aggcacagag aagtcagtca aggcatctct agatagagat

8701 gaaaacagaa tcatagagga ccctatgata cagctgttac cagaaaagct taggagagaa

8761 cttgccagac tgggattggc caaaatggaa gcttcagaac ttattcagca acctggtcct

8821 agtgatagct taagtagcct tgtggcacat aagctcatca caatgaactg tgccactgag

8881 gagtacaaag ctgaagtcat gaggttaaag caaactctaa cttctagaaa cgttctccat

8941 ggtctagctg gaggaatcaa agagttatca cttccaatat acacaatatt tatgaagtct

9001 tacttcttca aggacactgt ctttttag?? ?????????? ?????????? ??????????

9061 ?????????? ?????????? ?????????? ?????????? ?????????? ??????????

9121 ?????????? ?????????? ?????????? ???acatttg ataatacaca atgcgcacta

9181 tttgacagtt tatttgacga aaatttaagg tttgttgaag ttgtaagata tgaaggtggt

9241 atagttaatc tatgttatca gg???????? ?????????? ?????????? ??????????

9301 ?????????? ?????????? ??????aggg taagaggtta aggtcatgga argyggttgt

9361 gcagataata ttcttgaagc ccataaagca gt???????? ?????????? ??????????

9421 ?????????? ?????????? ?????????? ?????????? ?????????? ??????????

9481 ????????ar tgtmtrmkar rcmstccaca attgacatgg gtagcttagg aagagataga

9541 tttaarctat ctcagtttta ctcttcctta gttgagttgg taaatgaaat aaataatttg

9601 tcggaagctt tgaaaaagga aaaacgtatc ataaacttgg aagttgtgaa caaatttgcc

9661 aacaatctaa ctttgctatg caggcttgtt caacaagcaa gaagcaaagt gacctcrttc

9721 tatatgctga aaggttcgac tacaacaaat gaacccactg ttacagaact tgtgagcttt

9781 ggtattattg aagggaaata tttcgagtta aaagacatgg atgctgacac ctctgcttac

9841 agcctaaaat actggaaagt tctacagtgc atttcggcaa tatcagtcct gcctatttct

9901 gactctaata aaacaaacct actgaatagt ttcctaaact ggaaaccgag catttcagaa

9961 ttgtatgaaa ggtgtccact gagtaagaaa gagaagagag ttttggagga gtttaatggt

10021 aagacacttc ttgatttatt agcaagtgaa cttccaagta taaaagacga caaacaaaga

10081 aataacctag aagacatagt tgactttgtt agatcaccac taacactcct tagaaagaaa

10141 ccatacatcg gggtgacagc aaactttcag acatggggag atgggcagaa agatggaaga

10201 tttacatatt caagcagtag tggtgaggca acaggaatct tcatcagcac aaagctacat

10261 ttgtacctct ctcatggctc ccaggcctta ctgctagaag tggagaagaa agtgctcgct

10321 tggcttaaca agagaaggac agatgttgta acacaagagc agcactacta cttcatagac

10381 ctgctttgtg attttaggca tgtccctaaa aaggcaaacg atggaacaat taagggtgtt

10441 aaacccagta gaactgagcc aaagtattta gagttttatg atcctannnn naaggtgaag

10501 ataaagttgt taaaatcaaa gcaagcattc tcacagtaag gaaatgcggt ataaaagaca

10561 ttcttagtga accaaggctk gtgtggagta tgaatagttt aacaataata tatgatgaac

10621 agatcagtaa ggcatcattt catgataaca tattggagat aagaacacta ctagaccaag

10681 cactaggagt aaaagagaaa actgtgccgg aggcagttta cacnggctct aaagttacac

10741 trtctagaac aaaattcagc tcagacttgt tcttaaatag ccttctattg ttgcaccact

10801 tcttggaaca cactccttca tcagctattt gggaatcaca gacaaaatca gagatcatca

10861 agtacttaga cmwswmagac gggggcaaag gaaatcttaa atctatagca gacaaccttg

10921 caaagtcaac agtnnnnnnn nnnnnnnnnn nnnnngtatt ggaaggtgca gaagaagaaa

10981 agatatgtca ggtgttgaca actgctctag agaaagggga tctgacaatg aatgcatggc

11041 cagaggtgca aacctacctt gatgaaaatg ggatgcaaaa tattacttta gagtttttac

11101 agaaaggcct atcagactct tacagctggc agtttaagaa cacactcatt aagtccggac

11161 ctgccaggct tggaggattc agaggccttg taagtgcggt aggtgcagaa tcaataccga

11221 gatttcttgc tcctttaata gcagacggta agctattaag taaatctcta gcatgcttca

11281 tacaagccag gaactacctt tccaagtctg gtcttacaga cctagagttg gacggcatcg

11341 tatgtacaat aatctactgt gtacaagcga gacaaaagat aagaaaagaa cccaarttta

11401 gcccttcaac actgttaaaa atgtcttcta caagagcatt caaatcagct acggacaaat

11461 actcaatcaa ctttgaagtt gttgatgaaa aggtgttggt tatctgtaaa gtgaatactg

11521 ttaaaataga agaaataagg agan?????? ?????????? ?????????? ??????????

11581 ?????????? ?????????? ?????????? ?????????? ?????????? ?????????g

11641 tacatagcac tgtcttccaa gataacccta cggcagaagg agagttcttt ggaatacagt

11701 tgacatccaa gcacgcagaa agttgttcca ttgccggtct ctgggaactt tgctgtccag

11761 ggtgtcaatg gaggaagacg gatatgagta tagtagaatc tgtgatatct cttttacttg

11821 gtataaaaga caactcaggt atggacagaa ttgaaggtga cattcctctt gagtctgagg

11881 gtatccaaca aattacattt gccgcatcgn kggaggtcgc tag?

//

LOCUS Issyk-Kul_virus_PbGER_L_segment_First_assembly 12218 bp DNA linear UNA 12-NOV-2020

DEFINITION A new nucleotide sequence entered manually.

ACCESSION urn.local...1z-cn2v2ux

VERSION urn.local...1z-cn2v2ux

KEYWORDS .

SOURCE

ORGANISM .

FEATURES Location/Qualifiers

ORIGIN

1 ?????????? ?????????? ?????????? ?????????? ??????ataa gwgwywkgga

61 gaatatagct tgggagcagg tcactcagga gttcagcacg gctgtttgta acttccccat

121 agaggaagtt tttaacatcg accccatgct acctgatggc agatgtttct tccgagctat

181 ggccaagttc atgttcaact cttctgatga atggctcatt gtaaagcgcg cttgtattga

241 gtactctcgg caacactgga acaggtttct tgtctacact agactttatc ctagctctgc

301 tgattatgag agggacatca tgagggatga ctactgggga ggttctttgg aagcagaagt

361 cctgtctgat ttgtacaatc tgacaattca cttctgggtc acagatgacc atcagtggat

421 acaccatgtt caaaggtgga agaataacgc accccatatg agcatcaatc tgctattcaa

481 tcagaaccac tttgacctgc taaacctgaa agaatttgaa gaggatgagg aaaacctccc

541 tatactacct ttggaggaga aggtggatat tgctataaga tctgttgtaa cagattcaga

601 agatggttca gagccatctt tcttaagaga catctctgtt gacgactcaa cctctacctt

661 caaaagaaat ttagacttgg agttaatgga aatggcaaat atgaaagagc tacttttgac

721 caaaaagggg aataaaagag gttccaaaca aactgttgac atgaaagaga aggattttag

781 gaaggcagtt gaaaagggga agcaaattcc actgaaggct ggtaggattt taaatcatct

841 cttcagcatt cagatagaag gtgtaaatga aggagaactc ctgattctct accctaaaga

901 ctttaaccac aaacgacctg gtgcattctc aattactgac cttggacaca agttattgga

961 tggtagcaaa gaatttgcta aatccattag caaactaaca cttgtgatct ctggagagct

1021 gatgacgtac ttaaatagca gttacttaat taggatgtgc ttcccaggca ctggtctcag

1081 tcaaactcct gatttactac accctgctat taagattgac gcaactatct tggcatgcgc

1141 agttcttatt agctcctttc tttacaagag ttcgaatgac attaagagga aattccttac

1201 tcttgcttgt gaaagaatgg gtatcaacac atcaaagctt ttcaaagaaa tgcttaactt

1261 ctgcaatact gaaatgtatg agagtcttta caaggtagtt caacatttgt caggagtttt

1321 cttgaggaaa caagtggdkg wskyttmwra gtttttaaag cgtatgggcc cgcagagcaa

1381 actggcacta cagtgcatca accttgagtc ctgtga???? ???caatata agaagttact

1441 tcaggaacta agtgaaagag atatgaatga tgta?????? ?????????? ??????????

1501 ?????????? ?????????? ?????????? ?????????? ?????????? ??????????

1561 ?????????? ?????????? ?????????? ?????????? ?????????? ??????????

1621 ?????????? ?????????? ???????gtg cattattgaa ttctttatga ggaaaatgat

1681 ttttaaattt gtgagcctac aaggtaaggc ttattcaggt gcctcccttg gaaatttgtt

1741 agcatacgca cacaatcttt acttgtcgaa agatagtctt aatttaacta ttgaagat??

1801 ?????????? ?????????? ?????????? ?????????? ?????????? ??????????

1861 ?????????? ?????????? ?????????? ?????????? ?????????? ??????????

1921 ?????????? ?????????? ?????????? ?????????? ?????????? ??????????

1981 ?????????? ?????????? ?????????? ?????????? ?????????? ??????????

2041 ?????????? ?????????? ?????????? ?????????? ??????ccac taggtatgtc

2101 aattcagaca ttgttcccag aaaagtttga gatgtttctg gaaagaacac acctacaccc

2161 agaattcagg gatttggtgc ctgacttctt tttatataaa ccaaggataa ttaaggagga

2221 tacattcgaa gcatcaataa cgaggaaaga tgatgatcaa gtaargcatg ttgaccrtat

2281 tgaggctgta gaagatgcat cgacagtcaa actgacttca aagaaaagat ttcctttacc

2341 tgaagtaaac atacaggaag tcttatca?? ?????????? ?????????? ??????????

2401 ?????????? ?????????? ?????????? ?????????? ??acaggatg caagtaatga

2461 tgaagaggtc aaagttaatg aactatgtct aat??????? ?????????? ??????????

2521 ?????????? ?????????? ?????????? ?????????? ?????????? ??????????

2581 ?????????? ?????????? ?????????? ?????????? ?????????? ??????????

2641 ?????????? ?????????? ?????????? ?????????? ?????????? ??????????

2701 ?????????? ?????????? ?????????? ?????????? ?????????? ??????????

2761 ?????????? ?????????? ?????????? ?????????? ?????????? ??????????

2821 ?????????? ?????????? ?????????? ?????????? ?????????? ??????????

2881 ?????????? ?????????? ?????????? ?????????? ????gtgttg agggcgcaat

2941 actcacaaga gagtctgcag aggatatatt ggagtatgta ttagagaaca tggataaaat

3001 aataggtgag atgcaaaaga ccaaatttaa acacgaaata agcaagaacc agaggtctgc

3061 atttaaatta ttgattggct ggatgaatga ggacatactg agttacagat gcaatgactg

3121 tctcaagtca atccagagca atattagcaa gatagaaact gaagctgaag ttgcatctta

3181 cttagcacgg gagctgatgc cagtggaaaa ggattgctgt aaaagtmtgy ctcwtaywya

3241 tgaagttagc cctgtacagg gaagaatccc tagcctaaca aacatcaaac acaagccact

3301 tg???????? ?????????? ?????????? ?????????? ?????????? ??????????

3361 ?????????? ?????????? ?????????? ?????????? ?????????? ??????????

3421 ?????????? ?????????? ?????????? ?????????? ?????????? ??????????

3481 ?????????? ?????????? ?????????? aatatatgat gaagagaatg aaggtaaaag

3541 ttttaaaaag ggtggtctta a????????? ?????????? ?????????? ??????????

3601 ?????????? ?????????? ?????????? ?????????? ?????????? ??????????

3661 ?????????? ?????????? ?????????? ?????????? ?????????? ??????????

3721 ?????????? ?????????? ?????????? ?????????? ?????????? ??????????

3781 ?????????? ?????????? ?????????? ?????????? ?????????? ??????????

3841 ?????????? ?????????? ?????????? ?????????? ?????????? ??????????

3901 ?????????? ?????????? ?????????? ?????????? ?????????? ??????????

3961 ?????????? ?????????? ?????????? ?????????? ?????????? ???????gct

4021 tctcaagttt caatggtacc aggagttagt ctactatagc aaagtgtgtg agactttctt

4081 acaaagttgc agtgaatttc atagatcagg aattaaggtt ctaaggataa ggctcaccga

4141 taccaactta gttatagcgc tacctgct?? ?????????? ?????????? ??????????

4201 ?????????? ?????????? ?????????? ?????????? ?????????? ??????????

4261 ?????????? ?????????? ?????????? ?????????? ?????????? ??????????

4321 ?????????? ?????????? ?????????? ?????????? ?????????? ??????????

4381 ?????????? ?????????? ?????????? ?????????? ?????????? ??????????

4441 ?????????? ?????????? ?????????? ?????????? ?????????? ??????????

4501 ?????????? ?????????? ?????????? ?????????? ?????????? ??????????

4561 ?????????? ?????????? ?????????? ?????????? ?????????? ??????????

4621 ?????????? ?????????? ?????????? ?????????? ?????????? ??????????

4681 ?????????? ?????????? ?????????? ?????????? ?????????? ??????????

4741 ?????????? ?????????? ?????????? ?????????? ?????????? ??????????

4801 ?????????? ?????????? ?????????? ?????????? ?????????? ??????????

4861 ?????????? ?????????? ?????????? ?????????? ?????????? ??????????

4921 ?????????? ?????????? ?????????? ?????????? ?????????? ??????????

4981 ?????????? ?????????? ?????????? ?????????? ?????????? ??????????

5041 ?????????? ?????????a aagaagccaa gctggctcaa cgcagagtca ggctcctgtt

5101 aggcttgcca aacatcaaaa aaatgtctga atcttatgtt gactcaagag atgcagagga

5161 agaagaagac ttgagttcaa gttctaaaac tagctcaacg atgagttaca ctkcccgaaa

5221 cagtaaatcc agcattaaaa gtgctaggtc atttgcaaaa aggaggaacc cgcccacttc

5281 tatgttcggc ataagggcct caaaacagaa acctatgagt atagagagcg ggtttgtcat

5341 aactagggat gacaaaagag attatcaaca agcaataaca gacaaagtgg aggtc?????

5401 gtacagagca aataaagaat cagtctttaa agacatcatc ataataataa gagagaaccc

5461 aaaccacacc tttggcagct ttgagctaat acaagcatgt acagaaatag ctagagcaaa

5521 gtttcctcct gaagctatag acaaagccaa aagggatcct agaaattgga tcagtgtatc

5581 agaagtaact gaaacaacaa gcatcattgc ggaaccaaga gattttatat tcatcaagga

5641 tgcatacaga attattatag gtaatgaaaa caaaaaaatg gtaaagctat taagaggaaa

5701 gtttcagaga ctaggcatgt cttgtaaatc tgaaggtcat gacagggttc attgccaaga

5761 attactctcc acaataccat cattgacaga caagcaaaaa gatgatatta tcaaaggtat

5821 aattaaccct tccaaactta cattttacaa ttggcaagaa ctcatcaaaa aaggtgtgaa

5881 tgaagtgttg ttractaatg atggcaatta catattttgt tggttaaagt ccttgagtca

5941 gatggttaaa agtggtttaa gaggagaaat aaagaattta aagtacggac ctatgttgca

6001 aaaaggcaag ttgtgtccta aaagtaagat cttgagtagc gaggaacaca gtgccattaa

6061 gagatttatt gagtttttga agtcatgtac aaagkgagag gtaacagatg atataaacaa

6121 gtctgatata agtataacag atctaatctt ggcttgggtg aaattcacaa agtcctccaa

6181 attaagcaag acgatcatct ?????????? ???????cta agaaacctgt cagaaaagtt

6241 gaaaaaactt gatcaagatt ataatacact aataactttg aagaaagagc ttcctggtat

6301 aagcttttct aaagaagaaa tacttcttag acagggtgaa aaaatgcttt tgttggctca

6361 tgacaaagac ataatgcacc taactaactt gctcttctta atctgcttaa gctgtccatg

6421 gtgcattcag tacaagactt ttgaagctat aatgatgagg aatatggctg aagctgaagg

6481 atttaacttg ccaaaaagtg gaactaccat taatgagtta cacccagact ctgttataaa

6541 c????????? ?????????? ?????????? ?????????? ?????????? ??????????

6601 ?????????? ?????????? ?????????? ?????????? ?????????? ??????????

6661 ?????????? ?????????? ?????????? ?????????? ?????????? ??????????

6721 ?????????? ?????????? ???ctcgatc tgactttaaa tggactatca acctacttgc

6781 caatagcaac ttcgaggtga ctaagaaaat aacaggtaga agtgtgggtg agaagttgcc

6841 taggagtgtt agaagtaaag ttatctatga agttgttaaa ctagttgaca atactgagat

6901 ggctatattg caacaactgt ctttcactta tatactagat actaaccaca gattctttgc

6961 cgttcttgca ccaaaggcac aactgggtgg acatagggat cttcttgtgc aggkkgag??

7021 ??????gatg atacacgcca caactgagat gtttagcaga accttgctta gcactacgaa

7081 agatgatggg ctaacaaaca gcggcttgaa ggaatcaata ctcaatgcgg gtttggaggc

7141 cattaacacc atgaagctta accatggtaa agaaacttat cctcagtcag ggcagtttca

7201 gttctacaaa gtgtgctgca t????????a taatactaaa tggggtccaa tacattgttg

7261 ttcactgttc agtggaatga ?????????? ?????????? ?????????? ??????????

7321 ?????????? ?????????? ?????????? ?????????? ?????????? ??????????

7381 ?????????? ?????????? ?????????? ?????????? ?????????? ??????????

7441 ?????????? ?????????? ?????????? ?????????? ?????????? ??????????

7501 ?????????? ?????????? ?????????? ?????????? ?????????? ??????????

7561 ?????????? ?????????? ?????????? ?????????? ????catcta taatggctca

7621 tattatagag acactgataa agagatattt taagaaacac ?????????? ??????????

7681 ?????????? ?????????? ?????????? ??????gtgc atagttgctt ttggggtgtt

7741 aaacagagca ctctacaacc attatgaaga gagcttctgg gagcacatgt gcaggctgaa

7801 gaatttaatt tcaggatttt caagagcttg ccagatgaaa gattcagcta aaacacttgt

7861 ttctgactgc ttcttcaagt tttacagtga gtttatgatg tcacaaagaa taactccagc

7921 tgttataaaa ttcatattaa caggactcat taatagctca gtaacatcac ctttgagcct

7981 tatac????? ?????????? ?????????? ?????????? ?????????? ??????????

8041 ?????????? ????cactat tcaggcagca gatgttttac aaccacactg aaaattttgt

8101 tagaacctat ggccatttaa cattaggttc tgtgtcaagc tttggtaggc tgtatgtgcc

8161 caagtttagt aatctcattg gttcatcagt tgcacttgaa gatgctgaag aaattacaaa

8221 agcagctaac aatttaataa aagcaagtgt gcacttccct gaagcaattg gagacagctc

8281 tccatctagc aagacatctg atgacagtga ctcaatgtat aacgccacca cagattcagg

8341 tacagaagac acatcatcta ttggtagtgg ?????????? ?????????? ??????????

8401 ?????????? ?????????? ?????????? ?????????? ?????????? ??????????

8461 ?????????? ?????????? ?????????? ?????????? ??????atgt cgtatttgga

8521 caatggtgac tttagcgaag attgttacta ctcaaagaty ctggagtcat atagcgtcaa

8581 gaacaatgac tatcttggag atgatgagag gtcaccagaa ctagtgttga tgctgctgag

8641 gtctttgttg attgtcttga tttctggtta ctacaggact tttgccagtg aaggcacaga

8701 gaagtcagtc aaggcatctc tagatagaga tgaaaacaga atcatagagg accctatgat

8761 acagctgtta ccagaaaagc ttaggagaga acttgccaga ctgggattgg ccaaaatgga

8821 agcttcagaa cttattcagc aacctggtcc tagtgatagc ttaagtagcc ttgtggcaca

8881 taagctcatc acaatgaact gtgccactga ggagtacaaa gctgaagtca tgaggttaaa

8941 gcaaactcta acttctagaa acgttctcca tggtctagct ggaggaatca aagagttatc

9001 acttccaata tacacaatat ttatgaagtc ttacttcttc aaggacactg tctttttag?

9061 ?????????? ?????????? ?????????? ?????????? ?????????? ??????????

9121 ?????????? ?????????? ?????????? ?????????? ?????????? ??????????

9181 ????acattt gataatacac aatgcgcact atttgacagt ttatttgacg aaaatttaag

9241 gtttgttgaa gttgtaagat atgaaggtgg tatagttaat ctatgttatc agg???????

9301 ?????????? ?????????? ?????????? ?????????? ?????????? ??????????

9361 ?????????? ??ggtcatgg aaagtggttg tgcagataat attcttgaag cccataaagc

9421 agt??????? ?????????? ?????????? ?????????? ?????????? ??????????

9481 ?????????? ?????????? ?????????? ?????????a rtgtmtrmka ngmcmkccac

9541 aattgacatg ggtagcttag gaagagatag atttaarcta tctcagtttt actcttcctt

9601 agttgagttg gtaaatgaaa taaataattt gtcggaagct ttgaaaaagg aaaaacgtat

9661 cataaacttg gaagttgtga acaaatttgc caacaatcta actttgctat gcaggcttgt

9721 tcaacaagca agaagcaaag tgacctcrtt ctatatgctg aaaggttcga ctacaacaaa

9781 tgaacccact gttacagaac ttgtgagctt tggtattatt gaagggaaat atttcgagtt

9841 aaaagacatg gatgctgaca cctctgctta cagcctaaaa tactggaaag ttctacagtg

9901 catttcggca atatcagtcc tgcctatttc tgactctaat aaaacaaacc tactgaatag

9961 tttcctaaac tggaaaccga gcatttcaga attgtatgaa aggtgtccac tgagtaagaa

10021 agagaagaga gttttggagg agtttaatgg taagacactt cttgatttat tagcaagtga

10081 acttccaagt ataaaagacg acaaacaaag aaataaccta gaagacatag ttgactttgt

10141 tagatcacca ctaacactcc ttagaaagaa accatacatc ggggtgacag caaactttca

10201 gacatgggga gatgggcaga aagatggaag atttacatat tcaagcagta gtggtgaggc

10261 aacaggaatc ttcatcagca caaagctaca tttgtacctc tctcatggct cccaggcctt

10321 actgctagaa gtggagaaga aagtgctcgc ttggcttaac aagagaagga cagatgttgt

10381 aacacaagag cagcactact acttcataga cctgctttgt gattttaggc atgtccctaa

10441 aaaggcaaac gatggaacaa ttaagggtgt taaacccagt agaactgagc caaagtattt

10501 agagttttat gatcctaaag gtgaagataa agttgttaaa atcaaagcaa gcattctcac

10561 agtaaggaaa tgcggtataa aagacattct tagtgaacca aggctkgtgt ggagtatgaa

10621 tagtttaaca ataatatatg atgaacagat cagtaaggca tcatttcatg ataacatatt

10681 ggagataaga acactactag accaagcact aggagtaaaa gagaaaactg tgccggaggc

10741 agtttacac? ggctctaaag ttacactrtc tagaacaaaa ttcagctcag acttgttctt

10801 aaatagcctt ctattgttgc accacttctt ggaacacact ccttcatcag ctatttggga

10861 atcacagaca aaatcagaga tcatcaagta cttagacmws wmagacgggg gcaaaggaaa

10921 tcttaaatct atagcagaca accttgcaaa gtcaacagt? ?????????? ??????????

10981 ?gtattggaa ggtgcagaag aagaaaagat atgtcaggtg ttgacaactg ctctagagaa

11041 aggggatctg acaatgaatg catggccaga ggtgcaaacc taccttgatg aaaatgggat

11101 gcaaaatatt actttagagt ttttacagaa aggcctatca gactcttaca gctggcagtt

11161 taagaacaca ctcattaagt ccggacctgc caggcttgga ggattcagag gccttgtaag

11221 tgcggtaggt gcagaatcaa taccgagatt tcttgctcct ttaatagcag acggtaagct

11281 attaagtaaa tctctagcat gcttcataca agccaggaac tacctttcca agtctggtct

11341 tacagaccta gagttggacg gcatcgtatg tacaataatc tactgtgtac aagcgagaca

11401 aaagataaga aaagaaccca artttagccc ttcaacactg ttaaaaatgt cttctacaag

11461 agcattcaaa tcagctacgg acaaatactc aatcaacttt gaagttgttg atgaaaaggt

11521 gttggttatc tgtaaagtga atactgttaa aatagaagaa ataaggaga? ??????????

11581 ?????????? ?????????? ?????????? ?????????? ?????????? ??????????

11641 ?????????? ?????????? ?????gtaca tagcactgtc ttccaagata accctacggc

11701 agaaggagag ttctttggaa tacagttgac atccaagcac gcagaaagtt gttccattgc

11761 cggtctctgg gaactttgct gtccagggtg tcaatggagg aagacggata tgagtatagt

11821 agaatctgtg atatctcttt tacttggtat aaaagacaac tcaggtatgg acagaattga

11881 aggtgacatt cctcttgagt ctgagggtat ccaacaaatt acatttgccg catcgtggag

11941 gtcgctag?? ?????????? ?????????? ?????????? ?????????? ??????????

12001 ?????????? ?????????? ?????????? ?????????? ?????????? ??????????

12061 ?????????? ?????????? ?????????? ?????????? ?????????? ??????????

12121 ?????????? ?????????? ?????????? ?????????? ?????????? ??????????

12181 ?????????? ?????????? ?????????? ????????

//
